# Novel Insights into the Effect of Hyperforin and Photodynamic Therapy with Hypericin on Chosen Angiogenic Factors in Colorectal Micro-Tumors Created on Chorioallantoic Membrane

**DOI:** 10.3390/ijms20123004

**Published:** 2019-06-19

**Authors:** Martin Majerník, Rastislav Jendželovský, Marián Babinčák, Ján Košuth, Juraj Ševc, Zuzana Tonelli Gombalová, Zuzana Jendželovská, Monika Buríková, Peter Fedoročko

**Affiliations:** 1 Institute of Biology and Ecology, Faculty of Science, Pavol Jozef Šafárik University in Košice, Šrobárova 2, 041 54 Košice, Slovakia; martin.majernik@student.upjs.sk (M.M.); rastislav.jendzelovsky@upjs.sk (R.J.); marian.babincak@student.upjs.sk or burikovamonika@gmail.com (M.B.); jan.kosuth@upjs.sk (J.K.); juraj.sevc@upjs.sk (J.Š.); zuzana.gombalova@student.upjs.sk (Z.T.G.); zuzana.jendzelovska@upjs.sk (Z.J.); 2 Cancer Research Institute BMC, Slovak Academy of Sciences, Dúbravská cesta 9, 845 05 Bratislava, Slovakia

**Keywords:** angiogenesis, hypericin, photodynamic therapy, hyperforin, colorectal cancer, pro-angiogenic factors, experimental models, micro-tumors, quail embryo

## Abstract

Photodynamic therapy with hypericin (HY-PDT) and hyperforin (HP) could be treatment modalities for colorectal cancer (CRC), but evidence of their effect on angiogenic factors in CRC is missing. Convenient experimental model utilization is essential for angiogenesis research. Therefore, not only 2D cell models, but also 3D cell models and micro-tumors were used and compared. The micro-tumor extent and interconnection with the chorioallantoic membrane (CAM) was determined by histological analyses. The presence of proliferating cells and HY penetration into the tumor mass were detected by fluorescence microscopy. The metabolic activity status was assessed by an colorimetric assay for assessing cell metabolic activity (MTT assay) and HY accumulation was determined by flow cytometry. Pro-angiogenic factor expression was determined by Western blot and quantitative real-time polymerase chain reaction (RT-qPCR). We confirmed the cytotoxic effect of HY-PDT and HP and showed that their effect is influenced by structural characteristics of the experimental model. We have pioneered a method for analyzing the effect of HP and cellular targeted HY-PDT on pro-angiogenic factor expression in CRC micro-tumors. Despite the inhibitory effect of HY-PDT and HP on CRC, the increased expression of some pro-angiogenic factors was observed. We also showed that CRC experimental micro-tumors created on quail CAM could be utilized for analyses of gene and protein expression.

## 1. Introduction

In 1971, Judah Folkman stated the hypothesis that solid tumors are angiogenesis-dependent [1]. Until now, it was thought that angiogenesis promotes tumor growth and boosts the invasion and metastatic potential of those tumors [2]. The majority of human tumors persist in situ for months or years without neovascularization and consequently their size is limited to a small volume of a few cubic millimeters. The beginning of their development is associated with the switch to the angiogenic phenotype, which is linked to the recruitment of new blood vessels that support the growth of both angiogenic and non-angiogenic tumor cells [3]. High levels of effort and expectations have been expended to find anti-angiogenic compounds to treat colorectal cancer (CRC). CRC was the first malignancy for which anti-angiogenic treatment with bevacizumab in combination with 5-fluorouracil based chemotherapy was approved in 2004 (reviewed in [4]). Nowadays, there is huge evidence of agents with antiangiogenic potential as chemotherapeutics and also multikinase and topoisomerase inhibitors for CRC treatment approved mainly in combination with bevacizumab (reviewed in [5]). However, the effect of the current anti-angiogenic drugs is far from desirable and does not deliver acceptable curative results. A key effort is currently focused on the validation of biomarkers with potential anti-angiogenic effects in order to improve the cure for CRC. The results from clinical studies show that the presence of some angiogenic factors, like vascular endothelial growth factor A (VEGF-A), platelet-derived endothelial cell growth factor (PD-ECGF), and fibroblast growth factor 2 (FGF-2), in serum or tumor tissue is associated with the development of the disease in CRC patients (reviewed in [6,7]).

*Hypericum perforatum*, or St. John’s wort (SJW), extracts are extremely popular in Europe and the United States. St. John’s wort is used to treat mild to moderate depressive disorders or nervous unrest [8,9]. SJW contains a variety of bioactive constituents, of which flavonoids (rutin, quercitrin, hyperoside), phloroglucinols (hyperforin, adhyperforin), and naphtodianthrones (hypericin, pseudohypericin) are the most abundant [10,11]. There is some evidence that hyperforin (HP) and hypericin (HY), in combination with photodynamic therapy (HY-PDT), have anti-angiogenic effects and could inhibit tumor growth. The results of both treatment approaches are consistent with the fact that the antitumor effect was mainly observed only if both treatment protocols were focused on a vascular component of the tissues [12,13,14,15,16,17,18]. Besides this, the anti-angiogenic effect was, in both cases, affirmed in vivo using a chorioallantoic membrane (CAM) model [19,20]. In both treatment approaches, targeting cancer cells of the tumor mass evoked only a temporary inhibition of tumor growth. In HY-PDT, a regrowth of tumors in nasopharyngeal carcinoma was already noted in 24 h [21], and the same in bladder carcinoma in 72 h after therapeutic incidence [22].

We assume that it is very difficult to perform a rigorous vascular treatment in non-laboratory conditions (on non-controlled tumors) that affects even a small fraction of malignant cells in tumors. Besides the fact that angiogenesis is one of the hallmarks and key mechanisms for tumor progression in CRC, and that both treatment approaches have potentially anti-angiogenic effects, analyses have not yet been performed. Based on the above results and assumptions, we investigated the effect of HY-PDT and HP on selected CRC experimental models.

Additionally, the number of drugs with potential antitumor effects is growing, with only 10% approved for clinical use [23,24,25]. Therefore, we utilize 2D, 3D, and micro-tumor cell models for experimental analyses [26]. Using histological analyses, we are the first to show the relevance of micro-tumors created on quail CAM for molecular analyses focused on gene and protein expression. Moreover, the inhibition effect of both treatment modalities on 2D and 3D CRC experimental models was observed. We show that 3D cell models are more resistant to both treatment protocols than 2D cell models. Our results point to the fact that the effect of chosen therapies is influenced by the conditions of the cell environment.

## 2. Results

### 2.1. Dimensionality of Experimental Models as A Key Factor Affecting Accumulation Properties of HY and Metabolic Activity after HY-PDT and HP Treatment

**Figure 1:**
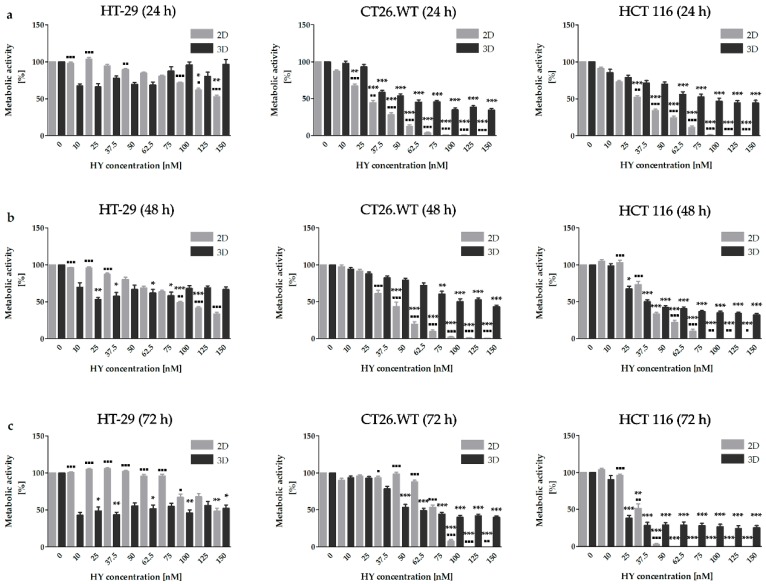
Metabolic activity in cell lines after treatment with HY-PDT. The 3D cell model is more resistant to HY-PDT in comparison to a 2D cell model. Metabolic activity was assessed in all experimental cell lines 24 h (**a**), 48 h (**b**), and 72 h (**c**) after irradiation by MTT assay. The results are expressed as the mean value ± SD of three independent experiments. The experimental groups cultivated in 2D and 3D cell models were compared with the control group (* *p* < 0.05, ** *p* < 0.01, *** *p* < 0.001). The experimental groups cultivated using 2D cell models were compared with experimental groups cultivated in 3D cell models (▪ *p* < 0.05, ▪▪ *p* < 0.01, ▪▪▪ *p* < 0.001).

**Figure 2:**
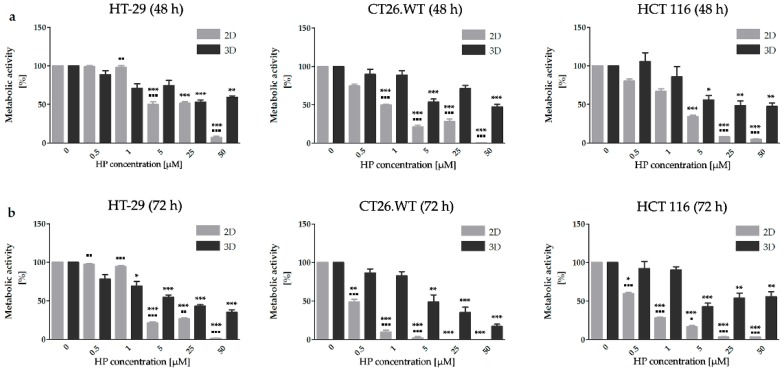
Metabolic activity in cell lines after treatment with HP. The 3D cell model is more resistant to HP treatment than the 2D cell model. Metabolic activity was assessed in all experimental cell lines 48 h (**a**) and 72 h (**b**) by MTT assay. The results are expressed as the mean value ± SD of three independent experiments. The experimental groups cultivated in 2D and 3D cell models were compared with the control group (* *p* < 0.05, ** *p* < 0.01, *** *p* < 0.001). The experimental groups cultivated in 2D cell models were compared with experimental groups cultivated in 3D cell models (▪ *p* < 0.05, ▪▪ *p* < 0.01, ▪▪▪ *p* < 0.001).

**Figure 3:**
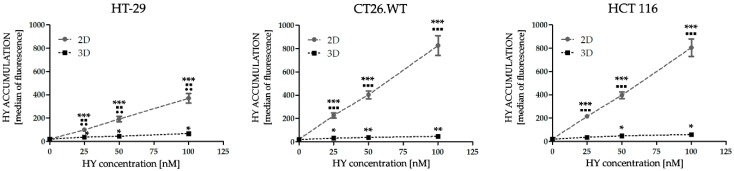
Comparison of intracellular accumulation of hypericin (HY) in 2D and 3D cell models. The incorporation of HY in all cell lines was analyzed 16 h after treatment. The results are expressed as the mean value ± SD of three independent experiments. The experimental groups cultivated in 2D and 3D cell models were compared with the control group (* *p* < 0.05, ** *p* < 0.01, *** *p* < 0.001). The experimental groups cultivated in 2D cell models were compared with experimental groups cultivated in 3D cell models (▪ *p* < 0.05, ▪▪ *p* < 0.01, ▪▪▪ *p* < 0.001). HT-29 cells cultivated in 2D and 3D cell model were compared with other two experimental cell lines (•• *p* < 0.01).

Metabolic activity was assessed as an overall signal resulting from the number of metabolizing cells (HT-29, HCT 116 and CT26.WT) and the intensity of their metabolism after exposure to HY-PDT for 24, 48, and 72 h (Figure 1) and in the HP treatment for 48 and 72 h (Figure 2). HY was applied in the concentration range of 0–150 nM and HP in the range of 0.5–50 µM. It is known that HY in the dark possesses zero or minimal toxicity [27,28,29,30,31]. Some biological effects on more sensitive cancer cell lines were described after the application of approximately eight times higher concentrations of non-photoactivated HY than our highest concentration was (150 nM). In relation to less sensitive cancer cells, after the application of approximately seventy times higher concentrations, some biological effect was detected [32,33,34,35]. More specifically, in connection with the growth factors analyses in the cancer cells treated with non-photoactivated HY, the effect was noticed only with the utilization of two hundred times higher concentration (30 µM), in comparison to our highest concentration [36]. When speaking of HY-PDT, these are the main reasons why the other authors also considered the non-treated cells or tumors to be a control [12,21,22,37,38]. Based on these facts, in our experiments utilizing HY-PDT non-photoactivated HY as dark control was did not use and we also considered the concentration of 0 nM HY as a control. In the 2D cell model, time- and dose-dependent inhibitory effects on the metabolic activity was observed in all cell lines after HY-PDT and HP treatment. Lower doses of HY demonstrated no or even a stimulatory effect on metabolic activity in the 2D cell models. HT-29 cells in both experimental models showed the highest resistance to HY-PDT treatment in all chosen time intervals. In 2D cell models, this can be associated with our observations from flow cytometry analyses, where there was the lowest accumulation of HY observed in the HT-29 cell line. In general, a significantly higher accumulation of HY was observed in the 2D cell models compared to the 3D cell models in all experimental cell lines. The significantly lower accumulation in the 2D cell models was observed in HT-29 in comparison to two other experimental cell lines in all HY treated groups. On the contrary, in the 3D cell models no significance was observed between the compared cell lines (Figure 3).

Similar results to those after HY-PDT treatment were observed after the treatment with HP (Figure 2). Overall, cells cultivated in 2D cell models were significantly more sensitive to treatment in comparison to the 3D cell models. In relation to HP treatment, a stimulatory effect on metabolic activity was observed in spheroids created from HCT 116 cells after the application of 0.5 µM HP. In the 2D cell models, HT-29 cells were the most resistant in both chosen times. On the other hand, if cells were cultivated in 3D cell models, the sensitivity of selected cell lines to HP treatment was more similar. Interestingly, in spheroids created from HCT 116 cells 5 µM concentration of HP significantly reduced the metabolic status of cells. In this case, more than 50% inhibition of metabolic status was observed 72 h after treatment.

### 2.2. Establishment of CRC Micro-Tumors on CAM

**Figure 4:**
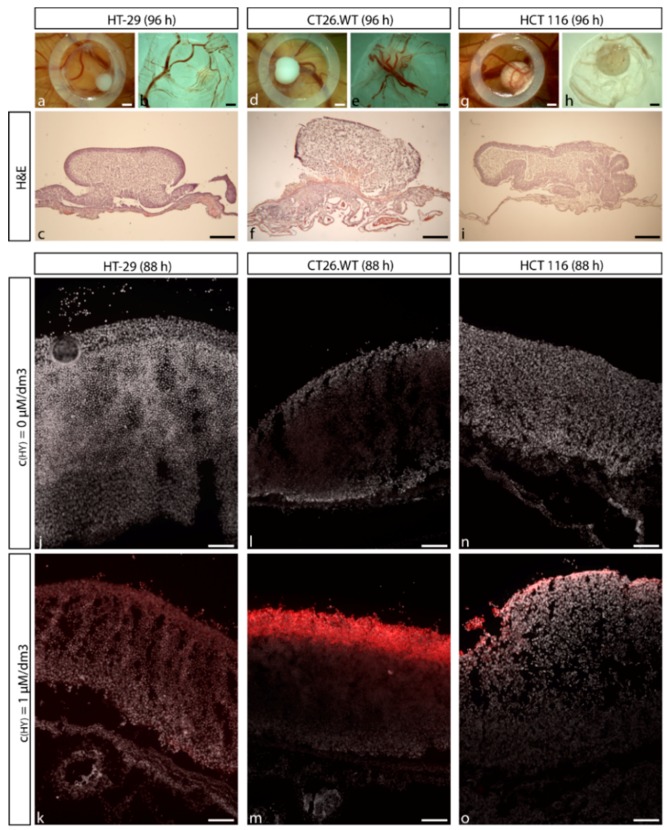
Micro-tumours produced from various cell lines imaged using light and fluoescence microscopy. Cells derived from CRC had a potential to create micro-tumors in CAM tissue. 25 × 10^5^ cells were topically applied into the area defined by a silicone ring. HT-29 (**a**–**c**), HCT 116 (**d**–**f**), and CT26.WT (**g**–**i**) cells created solid micro-tumors. View from above (**a**,**d**,**g**) and below (**b**,**e**,**h**). H&E staining of HT-29 (**c**), HCT 116 (**f**), and CT26.WT (**i**) micro-tumors was used to prove fully attached and vascularized micro-tumors. Non-treated (**j**,**l**,**n**) and HY treated (**k**,**m**,**o**) micro-tumors were analyzed by fluorescence microscopy for the detection of HY penetration into the tumor’s mass. Scale bar in (**a**,**b**,**d**,**e**,**g**,**h**) = 1 mm. Scale bar in (**c**,**f**,**i**) = 500 µm. Scale bar in (**j**–**o**) = 100 µm.

Since there is no data on protein and gene analyses of tumors isolated from CAM in recent literature, to our knowledge this is the very first attempt to verify the parameters of relevance in experimentally created tumors. Results from nucleo-cytoplasmic hematoxylin and eosin (H&E) staining showed that CAM primary germ layers were structurally deformed due to the consequences of formed micro-tumors. After 72 h from cell seeding on CAM, fully attached micro-tumors interconnected with CAM tissue were formed. Blood veins were dispersed through the tumor mass (Figure 4a–i). The detection of proliferating cells by anti-Ki-67 staining proved that experimental tumors possessed active proliferative status already at a time when selected secondary metabolites were topically applied on the created tumor (Figure S3). For HY-PDT treatment purposes, the accumulation properties of HY in experimental tumors were analyzed. We observed that topically applied HY penetrated into a tumor mass and was accumulated predominantly in the edges (Figure 4j–o). Based on these results, we concluded that experimentally created tumors had appropriate characteristics for the use in subsequent analyses focused on protein and gene expression changes after the selected treatment.

### 2.3. Effect of HY-PDT and HP on Expression of Pro-Angiogenic Factors in Early Stages after Treatment Incidence

Our aim was to analyze the effect of HP and HY-PDT targeted on the cellular portion of a CRC tumor mass on angiogenesis. Analyses were performed in the early stages, 24 h after the treatment. Analyses were performed on HT-29, HCT 116, and CT26.WT cell lines in both HY-PDT and HP treatment groups. Experiments focusing on gene and protein expression were performed on both 2D and micro-tumor cell models. Our first goal was to evaluate the reliability and applicability of these experimentally created micro-tumors for utilization in the following molecular analyses. We analyzed the gene expression of *PD-ECGF* [39,40,41], *FGF-2* [42,43,44], *VEGF-A* [45,46,47,48,49], and two alternative spliced forms of *PDGF-A* (*V1* and *V2*), as well as the protein expression of PD-ECGF, FGF-2, and VEGF-A, which are the key factors of angiogenesis in CRC. In the HY-PDT treated groups, we used 25 nM and 150 nM concentrations of HY, and in the HP treated groups 1 µM and 5 µM concentrations of HP. The evaluation of FGF-2 mRNA expression in HT-29 cells cultivated in 2D cell models was not possible, since the discrete product was not detected in quantitative real-time polymerase chain reaction (RT-qPCR).

#### 2.3.1. HY-PDT targeted at the Cellular Portion of CRC Micro-Tumors Upregulated Gene Expression of Pro-Angiogenic Factors

**Figure 5:**
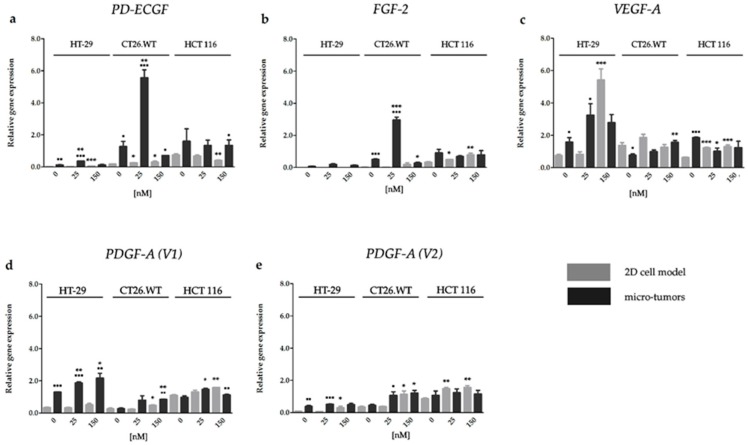
Effect of HY-PDT on the pro-angiogenic factor gene expression of *PD-ECGF* (**a**), *FGF-2* (**b**), *VEGF-A* (**c**), *PDGF-A* (*V1*) (**d**), and PDGF-*A* (*V2*) (**e**). The mRNA was quantified by RT-qPCR and normalized to the expression of phosphomannomutase 1 (*PMM1*). All results are expressed as the mean value of at least two independent experiments ± SD. The groups treated with HY-PDT were compared with untreated control (* *p* < 0.05, ** *p* < 0.01, *** *p* < 0.001). The experimental micro-tumors and cells cultivated in 2D cell model were compared with each other (▪ *p* < 0.05, ▪▪ *p* < 0.01, ▪▪▪ *p* < 0.001). Data were analyzed with one-way ANOVA with Tukey’s post-test or *t*-test.

**Figure 6:**
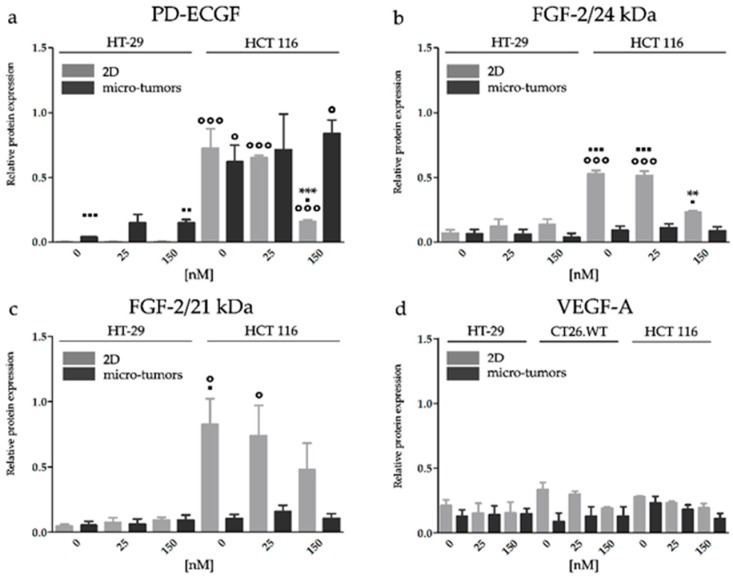
Effect of HY-PDT on the pro-angiogenic factors protein expression of PD-ECGF (**a**), FGF-2/24 kDa (**b**), FGF-2/21 kDa (**c**), and VEGF-A (**d**). Micro tumors are more resistant to HY-PDT than 2D cell models. The proteins were analyzed by Western blot. The effect of HY-PDT on PD-ECGF, FGF-2, and VEGF-A expression in cells cultivated in 2D and micro-tumor cell model was analyzed 24 h after PDT. β-actin served as a loading control. The groups treated with HY-PDT were compared with untreated control (** *p* < 0.01, *** *p* < 0.001). The experimental micro-tumors and cells cultivated in 2D cell models were compared with each other (▪ *p* < 0.05, ▪▪▪ *p* < 0.001), so were the particular cell lines (₀ *p* < 0.05, ₀₀ *p* < 0.01, ₀₀₀ *p* < 0.001). Data were analyzed with one-way ANOVA with Tukey’s post-test or *t*-test.

We observed that HY-PDT could induce gene expression of the majority of analyzed pro-angiogenic factors and that the expression level of genes (Figure 5) did not correlate with the expression level of proteins (Figure 6). As already mentioned, the cells cultivated in the 2D cell models were more sensitive to HY-PDT compared with the cells cultivated in the 3D cell models. Equally, the upregulation of gene expression after HY-PDT was observed mainly in the 2D cell models than in experimental micro-tumors. The decreased level of gene expression after HY-PDT was observed in the 2D cell models only by *PD-ECGF* in HCT 116 cells, and this result correlated with the protein expression level of this factor. On the other hand, HY-PDT induced expression of pro-angiogenic factors mainly in CT26.WT micro-tumors, where elevated levels of *PDGF-A* (*V1*), *PDGF-A* (*V2*), and *VEGF-A* were observed in the 150 nM HY-PDT experimental group. Moreover, the obvious upregulation of *PD-ECGF* and *FGF-2* was observed in the 25 nM HY-PDT experimental group. On the contrary, besides the fact that HY-PDT induced upregulation of pro-angiogenic factors mainly in CT26.WT micro-tumors, a decreased level of the *FGF-2* transcript after treatment was observed in the 150 nM HY-PDT experimental group. Also, only in this one case did we observe the decreased level of pro-angiogenic factor gene expression in experimental micro-tumors after HY-PDT. Furthermore, in experimental micro-tumors, the highest incidence of upregulation after HY-PDT was detected in *PDGF-A* (*V1*).

#### 2.3.2. HY-PDT Targeted at the Cellular Portion of CRC Micro-Tumors did not Upregulate Pro-Angiogenic Protein Expression

HT-29 cells cultivated in the 2D cell models showed a lower constitutive level of PD-ECGF than experimental micro-tumors. HY-PDT did not affect the factor expression pattern of the pro-angiogenic factors in both experimental models. A higher constitutive level of PD-ECGF was observed in HCT 116 micro-tumors than HT-29 micro-tumors, and the expression level was likewise unaltered after the treatment. A decreased level of PD-ECGF was observed only if HCT 116 cells were cultivated in a 2D cell model. Our observations from FGF-2 analyses in micro-tumors showed the opposite tendency to PD-ECGF. The HT-29 cell line showed very low levels of both FGF-2 isoforms in the 2D and micro-tumor cell models. Besides this, the expression levels of 24 and 21 kDa FGF-2 isoforms were unaltered after HY-PDT in both experimental models. In HCT 116 cells, we observed that in non-treated groups there was a constitutive level of both 24 and 21 kDa FGF-2 isoforms. A higher expression was noticed in the 2D cell models than in micro-tumors. A decreased level of the 24 kDa isoform was detected in HCT 116 2D cell models after the application of 150 nM HY. We observed that HCT 116 and HT-29 micro-tumors produce a very low amount of FGF-2 and the protein levels of both isoforms were unaltered after HY-PDT. We assume that the discrepancy between protein and gene expression in the 2D cell models (mainly in the 150 nM HY-PDT experimental group) could be caused by the massive damage caused to organelles associated with proteosynthesis. Whereas the higher concentration of HY could have toxic consequences for the cells cultivated in the 2D cell model, the same concentration of HY could have no effect on the in vivo micro-tumor CRC system. The expression level of VEGF-A was unaltered after HY-PDT treatment in all experimental groups (Figure 6 and Figure S1). Our results from HY-PDT, focused on the effect on pro-angiogenic factors, strongly indicate that the use of the appropriate experimental model is paramount for these types of analyses.

#### 2.3.3. Effect of HP on the Gene Expression of Factors Associated with CRC Angiogenesis

**Figure 7:**
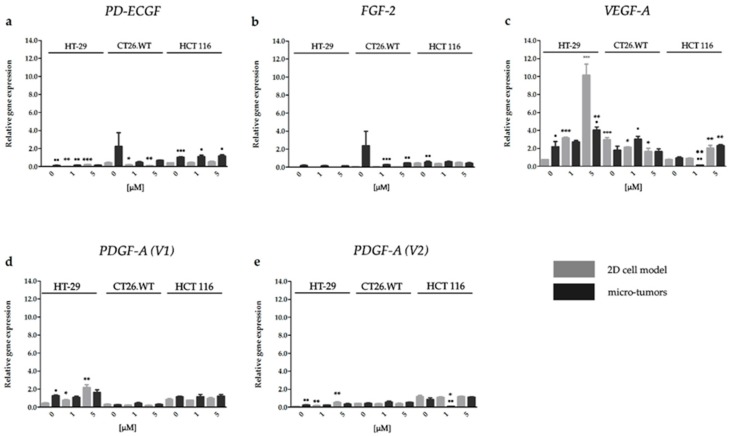
Effect of HP on pro-angiogenic factor gene expression of *PD-ECGF* (**a**), *FGF-2* (**b**), *VEGF-A* (**c**), *PDGF-A* (*V1*) (**d**) and *PDGF-A* (*V2*) (**e**). The mRNA was quantified by RT-qPCR and normalized to the expression of phosphomannomutase 1 (*PMM1*). All results are expressed as the mean value of at least two independent experiments ±SD. The groups treated with HP were compared with untreated control (* *p* < 0.05, ** *p* < 0.01, *** *p* < 0.001). The experimental micro-tumors and cells cultivated in 2D cell models were compared with each other (▪ *p* < 0.05, ▪▪ *p* < 0.01, ▪▪▪ *p* < 0.001). The non-treated groups of experimental micro-tumors similarly to non-treated groups of cells cultivated in 2D cell models were compared with each other in VEGF-A group (₀₀₀ *p* < 0.001). Data were analyzed with one-way ANOVA with Tukey’s post-test or *t*-test.

The results from RT-qPCR analyses revealed an increase in *VEGF-A* expression in HT-29 and HCT 116 cell lines cultivated in both experimental models after HP treatment. However, CT26.WT cells cultivated in 2D cell models presented a decreased level of *VEGF-A* in both treatment groups. On the other hand, we did not observe any changes in *VEGF-A* expression in CT26.WT experimental micro-tumors. The effect of cultivating conditions on the overall result of HP treatment was the most obvious in relation to gene expression in HT-29 cells cultivated in the 2D cell models, where we detected the upregulation in four of five analyzed pro-angiogenic factors. On the contrary, in HT-29 micro-tumors we observed alterations only in *VEGF-A*. In HCT, 116 micro-tumors treated with 1 µM HP, a downregulation of *VEGF-A* and *PDGF-A* (*V1*), were observed. The increased expression of *PD-ECGF*, *PDGF-A* (*V1*), and *PDGF-A* (*V2*) was observed in HT-29 cells only if they were cultivated in the 2D cell models. In experimental micro-tumors affected by HP treatment only the upregulation of *VEGF-A* expression was detected (Figure 7).

#### 2.3.4. HP Increased Angiogenic Potential of CRC Micro-Tumors by Upregulation of Proteins Associated with a Pro-Angiogenic Phenotype

Similar to HY-PDT, the analyses of the level of pro-angiogenic proteins were performed 24 h after the treatment. We observed that the HCT 116 cell line is more sensitive to HP treatment than the HT-29 cell line. HP treatment (at 5 µM) led to the increase in VEGF-A and 24 kDa FGF-2 in HCT 116 micro-tumors. On the contrary, non-treated HCT 116 cells cultivated in the 2D cell models presented higher constitutive levels of VEGF-A in comparison to the HCT 116 micro-tumors. Otherwise, we did not observe alterations in VEGF-A protein expression if the cells were cultivated in the 2D cell models. The expression level of PD-ECGF in HCT 116 cells was increased mainly after 5 µM HP, but significant alterations were observed only in the 2D cell models. A slight increase in PD-ECGF was also observed in HT-29 cell line cultivated in the 2D cell models.

**Figure 8:**
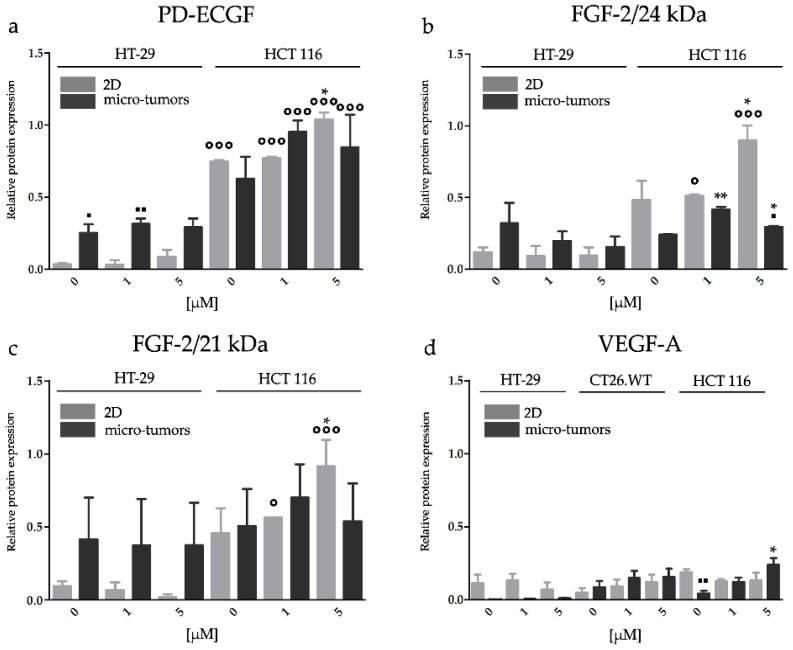
Effect of HP on the pro-angiogenic factors protein expression of PD-ECGF (**a**), FGF-2/24 kDa (**b**), FGF-2/21 kDa (**c**), and VEGF-A (**d**). HP could potentiate the expression of angiogenic proteins already 24 h after treatment. The proteins were analyzed by Western blot. The effect of HP on PD-ECGF, FGF-2, and VEGF-A expression in cells cultivated in 2D and micro-tumor cell models was analyzed 24 h after treatment. β-actin served as a loading control. The groups treated with HP were compared with untreated control (* *p* < 0.05, ** *p* < 0.01). The experimental micro-tumors and cells cultivated in 2D cell model were compared with each other (▪ *p* < 0.05), so were the particular cell lines (₀ *p* < 0.05, ₀₀₀ *p* < 0.001). Data were analyzed with one-way ANOVA with Tukey’s post-test or *t*-test.

HP treatment led to the increase of protein expression of both 24 and 21 kDa isoforms of FGF-2 in the HCT 116 2D cell models but only the 24 kDa isoform in experimental micro-tumors. The expression of PD-ECGF in the HT-29 cell line strongly depended on cultivation conditions, similar to the HY-PDT experimental group. HT-29 micro-tumors expressed a higher amount of PD-ECGF than the HT-29 cultivated 2D cell models, but HP treatment did not alter the expression of PD-ECGF in both experimental models. In addition, the conditions of the cellular environment could significantly modulate tumor response to HP treatment (Figure 8 and Figure S2).

## 3. Discussion

The key effort in cell biology research is to maintain the original phenotype of cells extracted from tissues and to mimic their native conditions [50,51]. Currently, the 2D cell model is conventionally utilized as an experimental model on a mass scale. Some evidence points to its limitations and suggests that alternative experimental methods should be utilized. These limitations include insufficient cell–cell and cell–extracellular matrix signaling, mechanisms that have key roles in a wide range of cellular functions [52,53]. Some essential signaling pathways could be missed or at least not fully developed in these experimental conditions [54]. Moreover, hypoxic conditions, one of the characteristic features of tumors, could dramatically affect the impact of some monoclonal antibodies [55,56], as well as chemotherapeutics [57].

The abovementioned reasons point to the utilization of more relevant experimental models [58,59]. It is very important to emphasize the fact that extracellular conditions could affect cellular phenotypes. In addition, the differences caused by micro environmental conditions affect the therapeutic effect of drugs in a significant manner [52,53]. The cells cultivated in 3D cell models are not inevitably more resistant to treatment than the cells cultivated in 2D cell models [57]. The cell reaction strongly depends on the characteristics of specific drugs and the cellular environment [60]. Moreover, there is much evidence that primary drug resistance is caused by inadequate tumor tissue drug penetration [61,62,63]. Some analyses showed that cultivation conditions in relation to in vitro and in vivo experimental models could significantly modulate the gene expression pattern of growth factors [64].

Moreover, the effect of HP on angiogenic factor expression was predominantly analyzed on 2D cell models, with the utilization of specific separately cultivated malignant or non-malignant cell lines [17,20,65,66]. These analyses represent an important tool for the clarification of basic mechanisms initiated by the treatment. However, in connection with the abovementioned information, the more complex experimental model systems, closely similar to real in vivo conditions, may produce different reactions to treatment.

In CRC, the angiogenic mechanism plays a very important role and it was the first malignancy for which anti-angiogenic treatment has been approved [4]. On the basis of the previously mentioned issues, and the fact that research on the anti-angiogenic potential of HY-PDT and HP has already been underway for more than ten years, our knowledge of the effect of these treatment modalities on CRC is deficient. Therefore, we decided to undertake these analyses with the conventionally utilized 2D cell model, the more relevant 3D cell model, and experimentally created micro-tumors (whose relevance was evaluated).

The cells cultivated in 2D cell models create only one homogenous layer. Typically, in 3D cell models we can recognize a necrotic core, surrounded by a viable rim, which consists of an inner layer of quiescent cells and an outer layer of proliferating cells [67]. There is a wide spectrum of evidence that spatial characteristics of 3D cell model affects mass transport through cellular barriers (reviewed in [68]). This information is closely related to our observations that HY accumulation in 3D was significantly lower than in the 2D cell model in all experimental cell lines. Correlated with these findings, we found that the 3D cell model had higher resistance to both treatment protocols than the 2D cell model. As we previously stated, the observed effect could be caused, mainly, by insufficient penetration of HY and HP deeper into the 3D cell model layers. Limited penetration and lower intracellular drug uptake could also be associated with low pH, which could induce net charge changes for some molecules. Moreover, the cells settled in deeper parts of the 3D cell model showed a proliferative non-active status, which was associated with higher resistance to treatment (reviewed in [69]). Molecular oxygen is one of the essential requirements for PDT (reviewed in [70]). We hypothesized that cells cultivated in 2D cell models were oxygenated in a sufficient way, and therefore PDT could have impacted total cell population. On the contrary, our results point to the fact that the presence of hypoxic regions in 3D cell model could also significantly decrease the potential of PDT in in vivo conditions.

Several analyses have been performed to evaluate microenvironment inside 3D cell models. The results indicate that this experimental model has analogous characteristics to avascular tissue or a tumor mass [71]. The angiogenic switch in CRC is already initiated in an early adenoma stage of malignancy development [72,73]. For these reasons, we assumed that a more complex experimental model, which includes vascular environment, should be used in the analyses focused on the angiogenic effects on CRC. CAM is a highly vascularized extra-embryonal membrane, with similar tissue responses to those in mammalian models (reviewed in [74]).

In relation to HY-PDT and HP, the anti-angiogenic effect of both treatment protocols was analyzed only on healthy vasculature of chick embryos [19,20]. There is some evidence of a possible utilization of CAM for xenograft tumor studies [75,76,77]. Moreover, we have assumed that micro-tumors created on CAM (as the highly reproducible experimental tumor model) could be an appropriate experimental model for molecular analyses conducted mainly in the early stages after the treatment incidence. Since there is no evidence of the utilization of CAM for these types of analyses, we assumed that the relevancy of the created micro-tumors must first be proven.

Histological analyses showed that all three experimental cell lines are able to create fully attached and vascularized micro-tumors with proliferative active status 72 h after topical application of the cells. Based on these results, we concluded that the 72 h micro-tumors were a suitable experimental model for further analyses focused on the protein and gene expression of factors associated with angiogenic progression in CRC.

The issue of tumor angiogenesis as one of the key factors important for tumor progression was first established by Judah Folkman in 1971 [1]. Up to the present, angiogenesis is one of the key hallmarks of cancer. There is much evidence that PDT is a potentially treated modality for CRC eradication (reviewed in [78]). The anti-angiogenic effects of HY-PDT and HP is still a highly debated problem. The possible anti-angiogenic effect is, in both cases, associated with the potential of both treatment modalities to destroy the vasculature of the tissues [12,13,14,17,19,20,65]. Some evidence shows that cellular targeted HY-PDT could improve the aggressive phenotype of tumors by increasing the expression of angiogenic factors in early stages after treatment incidence [21,22,79]. However, our knowledge about the effect of cellular targeted HY-PDT on CRC angiogenesis is very deficient.

The effect of HY-PDT was analyzed mainly on a bladder carcinoma [21,37], nasopharyngeal carcinoma [22,64,79], and fibrosarcoma [12,13]. The focal point of the present study concerns the effects of HY-PDT and HP on the expression of the key factors associated with CRC in the early stages after treatment incidence. As was demonstrated, HY-PDT focusing on the cellular component of the tumors can led to transient tumor growth inhibition with subsequent regrowth [12,13,14,22]. HY-PDT focusing on the cellular component of bladder carcinoma could increase the protein level of VEGF in affected tumors 24 h after HY-PDT [21]. Analyses conducted on nasopharyngeal carcinomas [22] showed the opposite effect, HY-PDT led to a decrease in the tumor and serum levels of VEGF after 24 h, followed by consecutively increasing expression levels 72 h after the treatment incidence [22]. Other authors stated a decreased level of VEGF in the serum of experimental animals with nasopharyngeal carcinomas 24 h after cellular targeted HY-PDT, in comparison with vascular targeted, but they did not state the level of expression in non-treated control tumors [79]. These results suggest that the effect of cellular targeted HY-PDT could be tumor specific. There is evidence that cellular targeted HY-PDT could induce a colon cancer regrowth after treatment incidence [14]. For the above mentioned reasons, our stated goal was to verify this phenomenon in CRC micro-tumors. Besides this, our next intention was to establish a new experimental model that was able to provide angiogenic research analyses.

Results from our experiments correlate with results of the above mentioned authors only partially [21,22,79]. The analyses of proteins did not confirm the changes of expression level in experimental micro-tumors (Figure 6). The alterations in pro-angiogenic protein synthesis were observed only if the cells were cultivated in a 2D cell model. Therefore, we assume that the angiogenic factors associated with potential tumor regrowth are not initiated in CRC before 24 h after treatment incidence, as it was observed in bladder carcinomas [21]. Research indicates that HY accumulates mainly in membranes of the endoplasmic reticulum, Golgi apparatus, lysosomes, and mitochondria [80,81,82,83]. Therefore, we assume that the unaltered status of protein expression observed in micro-tumors and a decreased protein expression in the 2D cell model (observed mainly in the 150 nM HY-PDT treatment group) could be also associated with damage to cell organelles participating in proteosynthesis. This hypothesis is supported by our observations from 2D cell models, where we detected a large discrepancy between protein and gene expression, which could be linked with a higher extent of intracellular organelle damage associated with the increased level of HY accumulation in 2D cell models compared to the experimental micro-tumors. Moreover, the increased level of gene expression, detected mainly in CT26.WT micro-tumors, could indicate the subsequent induction of protein synthesis in affected micro-tumors. Chen assumed that cellular targeted HY-PDT can induce direct cell death only in 30% of the tumor mass, with the progressive loss of cell viability in next 6 h after treatment incidence [13]. Relating to our results, it is possible that the fraction of HY-PDT treated surviving tumor cells with functional proteosynthetic apparatus could increase the expression of some pro-angiogenic factors as a self-protecting mechanism, which generally means that affected tumors could present a more aggressive phenotype.

Up to the present, there is some evidence for the anti-angiogenic potential of HP, another important secondary metabolite of *Hypericum perforatum*. However, the effect of HP on angiogenic mechanisms was examined in relation to non-malignant cells and tissues. Several studies showed that the effect of HP on endothelial cells (ECs) is cytostatic [65]. It can inhibit capillary formation [16,20,65], extracellular matrix (ECM) degradation [20], migration [20,65], the invasion potential of ECs [20], and affect the production or activity of matrix metalloproteinases [16,17,20,66]. Besides this, the antiangiogenic potential of HP on healthy vasculature with utilization in in vivo angiogenesis Matrigel sponge assays [65,66] and on healthy vasculature of chick CAM was described [20]. In relation to the effect of HP on the angiogenic mechanism in tumors and tumor associated vasculature, our knowledge is absolutely insufficient. There were some analyses performed on 2D cell models, which showed that HP could inhibit growth of breast fibrosarcoma, neuroblastoma, melanoma, prostate cancer, and colon cancer 16 h after treatment [20]; nevertheless, the effect was specific for each analyzed cell line. There is evidence that colon cancer is more sensitive in comparison to other types of tumors to HP treatment [17]. These findings are in correlation with our own, and the results focused on metabolic activity inhibition in all analyzed CRC cell lines. The inhibitory effect of HP was also shown in in vivo murine Kaposi sarcoma tumors, 28 days after a treatment incidence. However, there is missing evidence about fully cured tumors after HP treatment, even in long term effect observation studies [65]. In our opinion, it is important to verify if the treated tumors with temporally inhibited growth did not afterwards present a more aggressive phenotype induced by HP treatment.

Bearing the above in mind, it is important to note some evidence that shows HP treatment, in as low a concentration as 0.5 µM, can lead to significantly increased level of gene and protein expression of VEGF 6 h after treatment incidence in medulloblastoma and glioblastoma. However, the increased level of VEGF protein and gene expression after HP treatment was observed only in tumor cell lines with a low constitutive expression status of this growth factor. In a U87 glioblastoma cell line, the authors assumed a higher gene and protein constitutive level of VEGF and stated that HP treatment did not alter the gene expression pattern of this factor [15]. Other authors assumed the opposite findings after HP treatment in B lymphocytic leukemia cells. The level of VEGF protein expression was significantly decreased after treatment with a 1.8 µM concentration of HP 48 h after incidence. On the contrary, the level of FGF-2 was unaltered [16]. Tassone’s hypothesis [15] correlates with our experiments only in relation to *VEGF-A* expression in HT-29 and HCT 116 cells cultivated in 2D cell models. In the described cell lines, we detected lower levels of *VEGF-A* expression in non-treated control groups with a significant increase of *VEGF-A* after HP treatment. Conversely, CT26.WT cells cultivated in a 2D cell model expressed a significantly higher constitutive level of VEGF than the other two experimental cell lines, and HP treatment initiated the inhibition of *VEGF-A* expression after only a 1 µM HP application. These findings could be in agreement with other authors, who observed the inhibition potential of HP on VEGF secretion in leukemic B-cells [16], but we did not observe the alterations in VEGF-A protein expression if the cells were cultivated in a 2D cell model. In comparison to 2D cell models, we did not detect the alterations in *VEGF-A* expression in non-treated experimental micro-tumors created from chosen cell lines. In spite of these common characteristics, the effect of HP was cell specific. HT-29 and HCT 116 micro-tumors showed a similar reaction to HP treatment associated with the increasing gene expression. On the other hand, HP treatment in CT26.WT micro-tumors did not lead to increased *VEGF-A* expression.

Our results showed that not only VEGF-A, but also PD-ECGF and FGF-2 expression levels in CRC experimental models could be altered as a consequence of HP treatment. Moreover, VEGF-A, FGF-2, and PD-ECGF alterations were detected, not only on gene expression level, but also the protein level, both factors being upregulated. However, only in HCT 116 micro-tumors did we detect an increased level of VEGF-A and FGF-2.

Three experimental models were used for exploration purposes. All selected experimental methods proved that the effect of HY-PDT and HP treatment in relation to CRC angiogenesis is strongly affected by the structure and complexity of the experimental model. Moreover, the results from the flow cytometry and fluorescence microscopy analyses offered possible causes for the presented differences. The histological analyses proved that all selected CRC experimental cell lines could form a solid micro-tumor that is structurally interconnected with CAM tissue 72 h after the topical application of cells on CAM. Moreover, the results of the molecular analyses showed, not only to the potential utilization of micro-tumors in the research of CRC angiogenesis, that this experimental model is more convenient and relevant for this type of analysis.

## 4. Materials and Methods

### 4.1. Reagents

**Figure 9:**
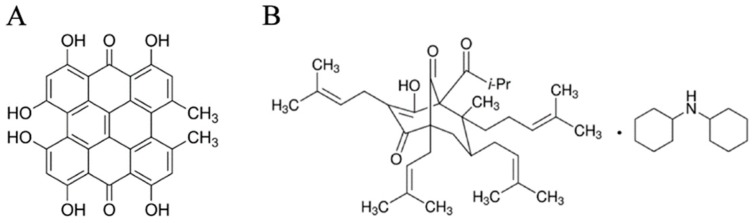
Structure of HY (**A**) and HP (**B**).

HY (CAS no.: 548-04-9, HPLC grade; AppliChem GmbH, Darmstadt, Germany) and HP (CAS no.: 238074-03-8, HPLC grade; Sigma-Aldrich, St. Louis, MO, USA) stock solutions (c = 2.6 mmol. dm^−3^ for HY and c = 5 mmol.dm^−3^ for HP) were prepared in dimethyl sulfoxide (DMSO, Lach-Ner, s.r.o., Neratovice, Czech Republic) and further diluted to working solutions that were freshly prepared immediately before addition to the cell culture (Figure 9). The final concentration of DMSO was less than 0.1% and did not influence the cytokinetic parameters. Since no significant differences in the response to the diluent were observed, these data are considered to be the control.

### 4.2. Cell Culture

HT-29, HCT 116, and CT26.WT cells were purchased from the American Type Culture Collection (ATCC, Rockville, MD, USA). HT-29 and CT26.WT cells were cultured in complete RPMI-1640 medium (Sigma-Aldrich, St. Louis, MO, USA) and HCT 116 cells in complete McCoy’s medium (PAN-Biotech GmbH, Aidenbach, Germany). Both cultivation media were supplemented with 10% fetal bovine serum (FBS; Biosera, Nuaille, France) and antibiotics (1% antibiotic-antimycotic 100× and 50 µg ml^−1^ gentamicin; Biosera, Nuaille, France) at 37 °C, 95% humidity, and in an atmosphere of 5% CO_2_.

### 4.3. Experimental Design

**Figure 10:**
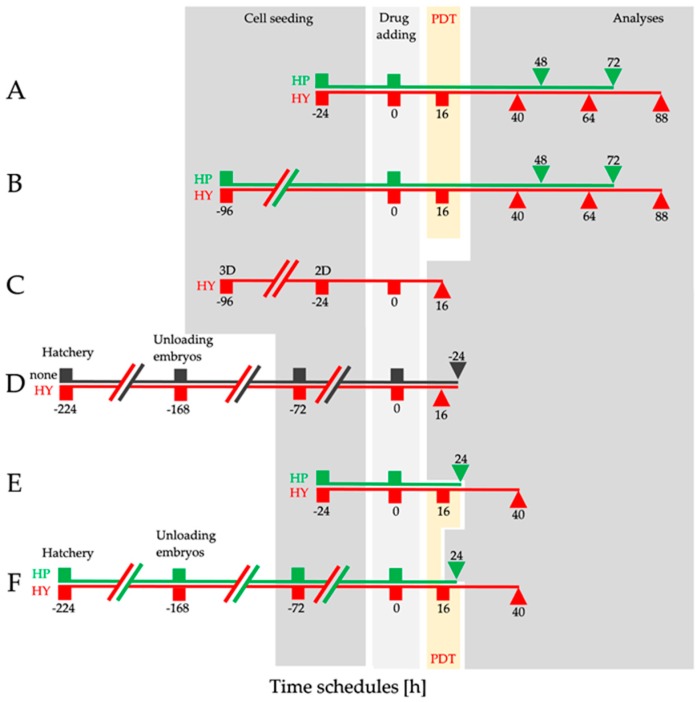
Experimental design. Time schedules **A** and **B**: MTT assays in the HY-PDT treatment group were performed 24, 48, and 72 h after PDT. HY was added 16 h before PDT (0 in time schedule). MTT assays on the HP treatment group were performed 48 and 72 h after HP addition (0 in time schedule). Time schedule **C**: The intracellular HY content was examined 16 h after HY addition. Time schedule **D**: Histological characteristics of micro-tumors were examined 72 h (0 in grey line—data not shown) and 96 h (24; grey line) after application of cells. HY was detected in experimental micro-tumors 16 h after topical application (red line). Time schedule **E** and **F**: Analyses of gene and protein expression were performed in the HY-PDT group 24 h after PDT (40; red line) and in the HP group 24 h after HP addition (24; green line).

For the experiments, cells were seeded in 96-well plates (MTT assay—2D and 3D cell model), in 6-well plates (flow cytometry analyses), or in 60 mm Petri dishes (Western blot, RT-qPCR), (all TPP, Trasadigen, Switzerland). All experiments were performed with HT-29, HCT 116, and CT26.WT cell lines. In 2D cell models, all cells were left to settle for 24 h before drug addition (Figure 10A,C,E). In 3D cell models, all cells were left to settle and to create spheroids for 96 h before drug addition (Figure 10B). In CAM micro-tumor models, were cells topically applied 72 h before drug addition (Figure 10F). Drug addition was performed at time 0 in all treatment protocols (Figure 10A–F). For MTT analyses in the HY-PDT treatment protocol, 10, 25, 37.5, 50, 62.5, 75, 100, 125, and 150 nmol.dm^−3^ HY was added to the cells. For the HP treatment, 0.5, 1, 5, 25, and 50 µmol.dm^−3^ HP was added (Figure 10A,B), for the intracellular HY content analyses 25, 50, 75, and 100 nmol.dm^−3^ HY was added (Figure 10C). For HY detection in micro-tumors analyses, 1 µmol.dm^−3^ HY was topically applied on micro-tumors. Both 2D and micro-tumor cell models were used for Western blot and RT-qPCR analyses. In the HY-PDT treatment protocol, 25 and 150 nmol.dm^−3^ HY was added and in the HP treatment protocol, 1 and 5 µmol.dm^−3^ HP was added.

### 4.4. HY Activation

Experimental cells cultivated in 2D, 3D, and as experimental micro-tumors in CAM tissue were treated with HY, as well as untreated controls. Cells of all chosen experimental cell lines were irradiated by irradiating device (a set of 2 × 25 LED, KVANT s.r.o, Bratislava, Slovakia) with a maximum emission range of 595 nm. Incorporated HY was activated by light at a total dose of 3.15 J·cm^−2^.

### 4.5. Spheroids (3D Cell Model) Formation Assay

Spheroids were generated using a liquid overlay technique, as previously reported [84]. Briefly, an agarose (SeaKem^®^ LE agarose, Lonza, Rockland, ME, USA) stock solution (0.8%) was prepared in dH_2_O and sterilized by autoclaving. Prior to the seeding of cells, the bottoms of 96-well plates were coated with 50 µL of agarose. Before use, the coated 96-well plates were exposed to ultraviolet germicidal irradiation for 0.5 h. After these procedures the plates were ready to use. Cell suspensions were prepared in such a way to allow the seeding of cells in a total volume of 100 µL. This particular seeding density was chosen for each cell line (HT-29: 2 × 10^4^/cells per well; HCT 116: 1 × 10^4^/cells per well; CT26.WT: 25 × 10^3^/cells per well), based on light microscopy observations. Using this protocol, spheroids were generated with a homogenous size distribution of similar diameter (Figure S4).

### 4.6. In vivo Experimental Micro-Tumor Creation

Fertilized quail eggs were purchased from a local hatchery and incubated for three days after breeding at 37.5 °C with 60% humidity. On the third day, each egg’s surface was cleaned using 70% ethanol. The egg-shell was perforated and the inner parts of the egg were placed into 6-well culture plates. Subsequently, embryos were incubated for four days at a temperature of 37 °C and 90% humidity. Afterwards, a sterile silicone ring with an inner diameter of 6 mm was used to define the experimental area. The space of the experimental area of each individual embryo was inoculated with 25 × 10^5^ cells resuspended in 50 µL of serum enriched medium. Subsequently, embryos were incubated for three days at a temperature of 37 °C and 90% humidity. After this time, experimental micro-tumors were created and prepared for use in experimental procedures.

### 4.7. MTT Assay

The MTT assays were performed as previously reported [85] to evaluate changes in the metabolic activity of cells that occurred as a consequence of HY-PDT or HP treatment. The cells were incubated with HY for 16 h. MTT (3-[4,5-dimethylthiazol-2-yl]-2,5-diphenyltetrazolium bromide) (Sigma-Aldrich, St. Louis, MO, USA) from a stock solution (5 mg mL^−1^) was added to the cells in a 96-well plate (TPP) 24, 48, and 72 h after HY-PDT and 48 and 72 h after HP treatment. The reaction was stopped after 4 h incubation at 37 °C and the insoluble formazan was dissolved by the addition of sodium dodecyl sulfate (SDS) at a final concentration of 3.3%. The absorbance (*λ* = 584 nm) was measured using a BMG FLUOstar Optima (BMG Labtech GmbH, Offenburg, Germany). Results were evaluated as percentages of the absorbance of the untreated control.

### 4.8. Intracellular Content of HY

Analyses of HY intracellular levels were performed 16 h after HY addition to 2D and spheroid cell models. The adherent cells were harvested by trypsinization and collected together with floating cells, washed in phosphate-buffered saline (PBS), and resuspended in Hank’s balanced salt solution (HBSS, Sigma-Aldrich, St. Louis, MO, USA). Six spheroids for every treated group were collected and dissolved using PBS-EDTA solution using an Eppendorf Thermomixer comfort (permanently mixed every 15 s, at 37 °C for 10 min) (Eppendorf, Hamburg, Germany). Subsequently, the samples were resuspended in HBSS. The analysis was performed (1 × 10^4^ cells per sample) using a BD FACSCalibur flow cytometer (Becton Dickinson, San Jose, CA, USA) with a 488 nm argon-ion excitation laser. Fluorescence was detected via a 585/42 nm band-pass filter (FL-2). The intensity of HY fluorescence in the cells was quantified using FlowJo software (Tree Star Inc., Ashland, OR, USA).

### 4.9. Western Blot Analysis

For Western blot analyses the cells were cultured in 2D cell models and in the in vivo CAM system of quail (*Coturnix japonica*) aviary embryos, where the experimental micro-tumors were created. HT-29, HCT 116, and CT26.WT cells cultured in 2D cell models were treated and harvested in line with the experimental design (Figure 10E). In the CAM model, experimental micro-tumors were isolated by micro scissors in line with the time schedule (Figure 10F). Then, cells and experimental micro-tumors in both experimental models were washed twice using ice-cold PBS and lysed for 10 min on ice in a lysis buffer (100 mM Tris-HCL, pH 7.4; 1% SDS, 10% glycerol), which was supplemented with a protease inhibitor cocktail (P2714; Sigma-Aldrich, St. Louis, MO, USA). During the lysis interval, every experimental micro-tumor was mechanically disrupted with Kimble motor 749540-0000 (Kimble, Mainz, Germany). The cell lysates were sonicated at 30% power of a Bandelin Sonoplus HD2070 (Bandelin Electronic, Berlin, Germany) on ice and were subsequently centrifuged. Protein concentrations were determined using a detergent compatible protein assay (Bio-Rad Laboratories, Hercules, CA, USA). Samples were diluted in equal amounts (30 µg of proteins), supplemented with sample buffer (12 mM Tris-HCL, pH 6.8; 0.02% bromophenol blue; 2.88 mM β-mercaptoethanol; 0.4% SDS; 5% glycerol), separated with 15% SDS-polyacrylamide gel (acrylamide/bis solution, 3.75:1), and transferred (wet transfer, 70 min) onto a polyvinylidene difluoride membrane (PVDF; Bio-Rad Laboratories) in a transfer buffer (10% methanol, 192 mM glycine, 25 mM Tris). The unoccupied membrane binding sites on the blots were incubated with 5% non-fat milk or 2.5% BSA in Tris buffered saline (TBS) (20 mM Tris-HCL, pH 7.6; 150 mM NaCl; 0.05% TWEEN 20, pH 7.4) for 1 h at room temperature (RT). Subsequently, the PVDF membrane blots were incubated with the following primary antibodies overnight at 4 °C: anti-VEGF-A (VEGF-A, mouse monoclonal, ab1316, Abcam); anti-FGF-2 (FGF-2, mouse monoclonal, Cat. no.: sc-74412, Santa Cruz Biotechnology, Dallas, TX, USA); anti-PD-ECGF (PD-ECGF, mouse monoclonal, Cat. no.: sc-47702, Santa Cruz Biotechnology, Dallas, TX, USA), followed by incubation with horseradish peroxidase-conjugated (HRP) secondary antibody goat anti-mouse IgG-HRP, (dilution: 1:5000, Cat. no.: 31436, Pierce Biotechnology, Rockford, IL, USA) for 1 h at RT. β-actin (A5441, mouse monoclonal, Sigma-Aldrich, St. Louis, MO, USA) was used as the loading control. The protein expression of FGF-2 and PD-ECGF was detected just in the experimental cells of human origin. The densitometry of proteins was evaluated using ImageJ software (NIH, Bethesda, MD, USA). Relative protein levels were normalized to the expression of β-actin.

### 4.10. Real Time RT-PCR (RT-qPCR)

The level of expression of the target genes (pro-angiogenic factors) and the reference gene was quantified by real time RT-qPCR with SYBR Green I detection of the amplicons. The cDNA template for RT-qPCR was prepared from total RNA by RevertAid™ M-MuLV Reverse Transcriptase (RevertAid First Strand cDNA Synthesis Kit, Thermo Fisher Scientific Inc, Waltham, MA, USA) and a mix of anchored-dT and random hexamer primers (NEB, USA). Total RNA was extracted by TRI Reagent^®^ (MRC Inc, Cincinnati, OH, USA) according to the manufacturer’s instructions. HT-29, CT26.WT, and HCT 116 cells cultivated in 2D cell models were harvested in line with the experimental design (Figure 10E). Whole micro-tumors from each treatment were isolated by micro scissors in line with experimental design (Figure 10F). All samples were collected in TRI Reagent and stored at −80 °C until RNA extraction. The samples were resuspended and mechanically ground directly in TRI reagent with sterile disposable pellet pestles attached to a Kimble motor 749540-0000 (Kimble, Mainz, Germany). RNA integrity was checked by agarose gel electrophoresis and the amount of RNA/µL was determined using a BioSpec nano-spectrophotometer (Shimadzu, Kyoto, Japan). Only samples with distinct 28S/18S RNA bands were used for cDNA transcription. Gene specific primers (Table 1) were designed by PrimerBLAST [86], hosted at the NCBI web page. The properties for primer design were set to be human (for HT-29, HCT 116 cell culture) or mouse specific (for CT26.WT). The primers either spanned two neighboring exons or bound to different exons. Primer pairs with no secondary structures and the best delta G values were chosen by Unipro UGENE v1.20.0 software [87].

RT-qPCR experiments were performed using a CFX96 Touch Real Time PCR Detection System (BioRad Laboratories, Hercules, CA, USA). For amplification reactions, Xceed qPCR SG Mix, Lo-Rox (IAB, Prague, Czech Republic), 0.5 μM For/Rev primers and 15 ng of RNA/cDNA were used. All samples were analyzed in duplicate. The amount of the target and reference gene transcripts, as well as the PCR efficiency, was evaluated according to a standard curve. The resulting values of target gene expression were normalized to the expression of *PMM1*, which was a gene shown to be an accurate internal reference control for the comparison of HT-29 and HCT 116 cultivated in 2D cell models and as experimental micro-tumors [88].

### 4.11. Tissue Processing

For H&E staining and anti-Ki-67 immunofluorescent labeling, the experimental micro-tumors were carefully dissected from quail CAM. Tumors were fixed in 4% paraformaldehyde (PFA) overnight and cryopreserved in 30% sucrose for 48 h. After cryoprotection, tumors were sectioned using cryostat Leica CM 1850 (Leica, Wetzlar, Germany) to 10 µm thick transverse sections for histological staining or 20 µm thick sections for immunolabeling. For HY detection, experimental micro-tumors were directly sectioned after isolation from CAM tissue.

### 4.12. Histology, Immunolabeling, and HY Detection

For histological examination, 10 µm thick sections were mounted on poly-L-lysine coated glass and stained according to standard H&E staining protocol, dehydrated, and mounted with Vectamount^®^ Permanent mounting medium (Vector Laboratories, Cat. no.: H-5000, Burlingame, CA, USA). For anti-Ki-67 immunofluorescent labeling, 20 µm thick sections were washed in 0.1 M PBS. Nonspecific protein activity was blocked with 5% normal donkey serum (NDS, RRID:AB_2337258, Jackson Immunoresearch, Cambridgeshire, UK) in 0.1 M PBS with 0.3% Triton X-100 for 30 min at RT. Sections were incubated with anti-Ki-67 antibody (dilution: 1:500, rabbit monoclonal, Cat. No.: ab16667, Abcam, Cambridge, UK) diluted in 0.1 M PBS, containing 1% NDS and 0.3% Triton X-100, overnight at 4 °C (Figure S3). After incubation with the primary antibody, sections were washed in 0.1 M PBS and incubated with anti-rabbit secondary antibody (dilution: 1:500, AlexaFluor488 donkey anti-rabbit IgG, Cat. No.: ab150073, Abcam, Cambridge, UK), diluted in 0.1 M PBS with 1% NDS and 0.3% Triton X-100 for 2 h at RT. After incubation with the secondary antibody, the sections were washed in 0.1 M PBS, counterstained with DRAQ5 (dilution: 1:1000, Cat. no.: 4084, Cell Signaling Technology, Leiden, Netherlands) for nuclear staining for 30 min at RT, and mounted with ProLong^®^ Gold anti-fade mounting medium (Cat. no.: 9071, Cell Signaling Technology). For HY detection, control non-treated and HY treated (c = 1 µmol.dm^−3^) micro-tumors were sectioned on 20 µm thick sections and mounted with ProLong^®^ Gold anti-fade mounting medium (Cell Signaling Technology) directly after isolation from CAM in dark conditions.

### 4.13. Brightfield and Confocal Microscopy

The H&E stained sections were analyzed with a brightfield microscope (BX41, Olympus Corp., Tokyo, Japan) and processed by Quick-PHOTO Micro 2.2 software (Promicra s.r.o., Prague, Czech Republic). Fluorescently labeled sections were analyzed using a Leica TCS SP5X confocal microscope system (Leica, Wetzlar, Germany), (10x magnifying objective lens were used for analyses of HY and 40x magnifying objective lens were used for analyses of proliferating cells with anti-Ki-67 antibody), (Leica Microsystems, Mannheim, Germany). The single optical sections, or z-stacks, were captured in LAS AF software (Leica Microsystems) and analyzed in ImageJ (NIH). All of the image manipulations were limited to brightness/contrast and were performed in a standardized manner. The images were assembled into figures using Adobe Illustrator (Adobe Systems, San Jose, CA, USA).

### 4.14. Statistical analysis

Data were analyzed using a one-way ANOVA with Tukey’s post-test or *t*-test and are expressed as the mean ± standard deviation (SD) of at least two independent experiments. Significance levels are indicated in the legend for each particular figure.

## Figures and Tables

**Table 1:** Nucleotide sequences of primers used for RT-qPCR.

Gene (GenBank Ref.seq.)	Cell lines	Primer Name	Sequence	Ta and Product Length
*hPD-ECGF* (NM_001113755)	HT-29, HCT 116	hTymp_for	TCAATGTCATCCAGAGCCCAG	60 °C 188 bp
		hTymp_rev	CCCCTCCACGAGTTTCTTACT	
*mPD-ECGF* (NM_138302)	CT26.WT	mTymp_for	ATCGCACAGCCCTAAGTCTC	58 °C 71 bp
		mTymp_rev	CCCTAGAGCCAGTAGCATCG	
*hVegfA* (NM_001025366)	HT-29, HCT 116	hVegfA_for	AGGAGGGCAGAATCATCACGA	60 °C 138 bp
		hVegfA_rev	ACACAGGATGGCTTGAAGATGT	
*mVegfA* (NM_001287056)	CT26.WT	mVegfA_for	TCTTCAAGCCGTCCTGTGTGC	61 °C 127 bp
		mVegfA_rev	CTTTGGTGAGGTTTGATCCG	
*hPdgfA* (V1) (NM_002607)	HT-29, HCT 116	hPdgfA_for1	AAGCAGCCAACCAGATGTGA	61 °C 133 bp
		PdgfA_rev1/2	GGAGGAGAACAAAGACCGCA	
*mPdgfA* (V1) (NM_008808)	CT26.WT	mPdgfA_for1	GGAGGAGACAGATGTGAGGTG	61 °C 134 bp
		PdgfA_rev1/2	GGAGGAGAACAAAGACCGCA	
*PdgfA* (V2) (NM_033023)	HT-29, HCT 116, CT26.WT	PdgfA_for2	AGGACACGGATGTGAGGTGA	60 °C 128 bp
		PdgfA_rev1/2	GGAGGAGAACAAAGACCGCA	
*FGF2* (NM_001361665)	HT-29, HCT 116, CT26.WT	FGF2_for	GAAGAGCGACCCTCACATCAA	61 °C 265 bp
		FGF2_rev	CTGCCCAGTTCGTTTCAGTG	
*PMM1* (NM_002676)	HT-29, HCT 116, CT26.WT	PMM1_for	GCTCGCCAGAAAATTGACCCT	61 °C 177 bp
		PMM1_rev	ATACTGCACCGTCCCGTTCT	

## Supplementary Materials

Supplementary materials can be found at https://www.mdpi.com/1422-0067/20/12/3004/s1.

## Abbreviations

CAM	Chorioallantoic membrane
CRC	Colorectal cancer
DMSO	Dimethyl sulfoxide
ECM	Extracellular matrix
ECs	Endothelial cells
FGF-2	Fibroblast growth factor 2 (protein)
*FGF-2*	Fibroblast growth factor 2 (gene)
H&E	Hematoxylin and eosin
HP	Hyperforin
HY	Hypericin
HY-PDT	Photodynamic therapy with hypericin
PD-ECGF	Platelet derived endothelial cell growth factor (protein)
*PD-ECGF*	Platelet derived endothelial cell growth factor (gene)
PDGF-A	Platelet derived growth factor subunit A (protein)
*PDGF-A* (*V1*)	Platelet derived growth factor subunit A isoform 1 (gene)
*PDGF-A* (*V2*)	Platelet derived growth factor subunit A isoform 2 (gene)
*PMM1*	Phosphomannomutase 1 (gene)
RT-qPCR	Quantitative real-time polymerase chain reaction
SJW	St. John’s wort
VEGF-A	Vascular endothelial growth factor subunit A (protein)
*VEGF-A*	Vascular endothelial growth factor subunit A (gene)
