# Supplementary material for: Novel Insights into the Effect of Hyperforin and Photodynamic Therapy with Hypericin on Chosen Angiogenic Factors in Colorectal Micro-Tumors Created on Chorioallantoic Membrane
Source: Int J Mol Sci. 2019 Jun 19;20(12):3004. doi: 10.3390/ijms20123004 (PMC6627608; doi:10.3390/ijms20123004)
Supplement: Supplementary file 1 [file ijms-20-03004-s001.pdf]

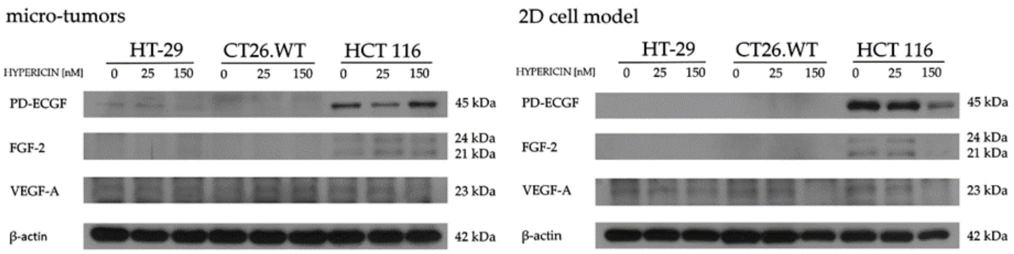

Figure S1. Effect of HY-PDT on pro-angiogenic factors protein expression of PD-ECGF, FGF-2/24 kDa, FGF-2/21 kDa, and VEGF-A (d). Micro tumors are more resistant to HY-PDT than 2D cell models. The effect of HY-PDT on PD-ECGF, FGF-2, and VEGF-A expression in cells cultivated in 2D and micro-tumor cell model was analyzed 24 h after PDT.

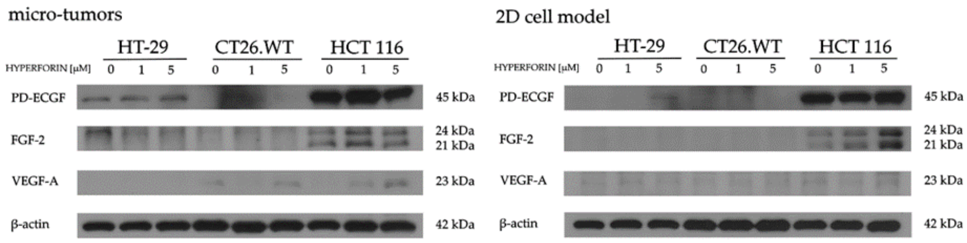

Figure S2. Effect of HP on pro-angiogenic factors protein expression of PD-ECGF, FGF-2/24 kDa, FGF-2/21 kDa, and VEGF-A (d). HP could potentiate expression of angiogenic proteins already 24 h after treatment. The effect of HP on PD-ECGF, FGF-2, and VEGF-A expression in cells cultivated in 2D and micro-tumor cell model was analyzed 24 h after treatment. β-actin served as a loading control. One representative picture is presented.

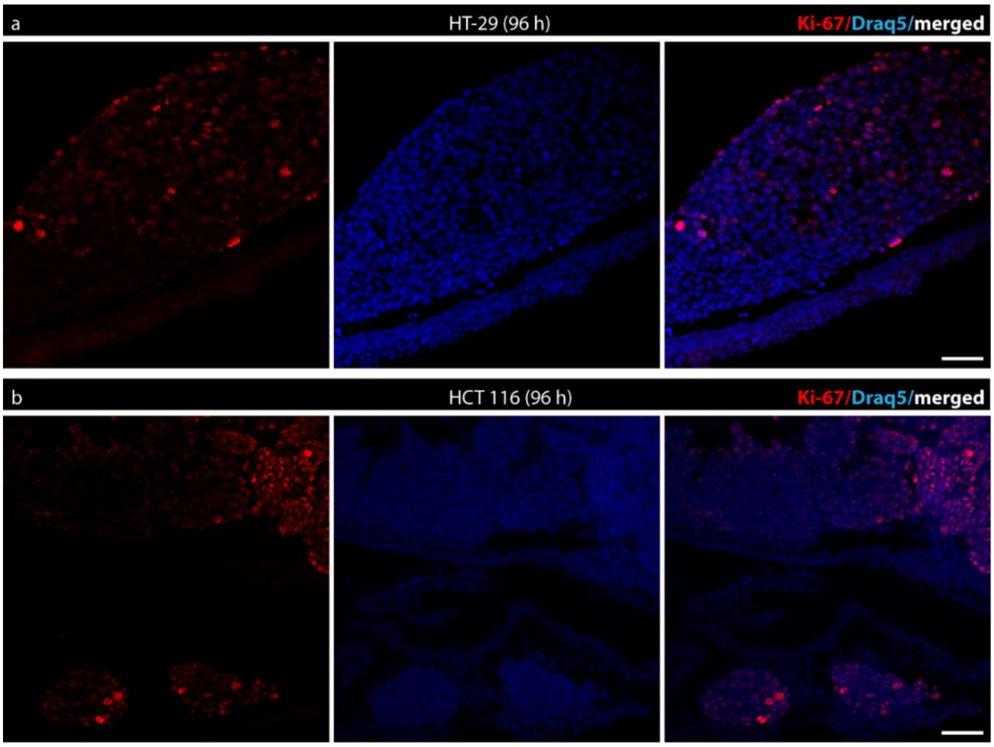

Figure S3. Detection of proliferative active cells in HT-29 (**a**) and HCT 116 (**b**) micro tumors. Dividing cells are viewed as red fields in the picture (marked with anti-Ki-67 antibody). Scale bar = 50 μm.

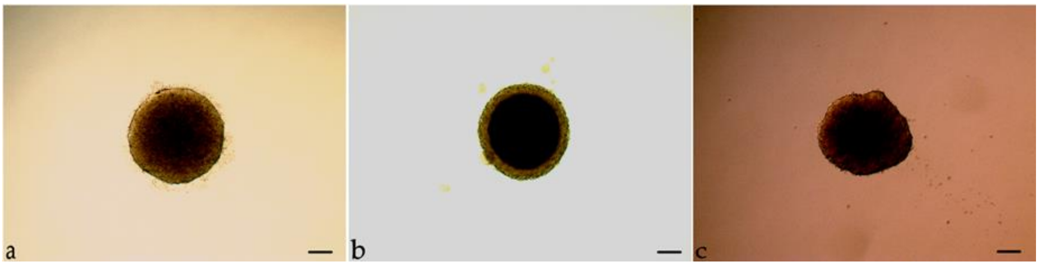

Figure S4. Spheroids created from various cell lines, HT-29 (**a**), CT26.WT (**b**), and HCT 116 (**c**). Images of spheroids were taken 96 h after cells seeding using light microscopy (Motic AE 2000 equipped with Moticam 5.0 MP color digital camera). Feret diameter of spheroids: HT-29 (**a**) = 785 μm, CT26.WT (**b**) = 774 μm, and HCT 116 (**c**) = 715 μm. Scale bar **a**–**c** = 200 μm.
